# Obstacle avoidance in bumblebees is robust to changes in light intensity

**DOI:** 10.1007/s10071-020-01421-z

**Published:** 2020-08-09

**Authors:** Emily Baird

**Affiliations:** Department of Zoology, Stockholm University, Stockholm, Sweden

**Keywords:** Insect, Flight, Obstacle avoidance, Light intensity, Bumblebee, Vision

## Abstract

Flying safely and avoiding obstacles in low light is crucial for the bumblebees that forage around dawn and dusk. Previous work has shown that bumblebees overcome the limitations of their visual system—typically adapted for bright sunlight—by increasing the time over which they sample photons. While this improves visual sensitivity, it decreases their capacity to resolve fast motion. This study investigates what effect this has on obstacle avoidance in flight, a task that requires the bees to reliably detect obstacles in the frontal visual field and to make a timely diversion to their flight path. In both bright and dim light, bumblebees avoided the 5 cm diameter obstacle at a consistent distance (22 cm) although in dim light they approached it more slowly from a distance of at least at least 80 cm. This suggests that bumblebees have an effective strategy for avoiding obstacles in all light conditions under which they are naturally active, and it is hypothesised that this is based on a time-to-contact prediction.

**Electronic supplementary material:**

The online version of this article (10.1007/s10071-020-01421-z) contains supplementary material, which is available to authorized users.

## Introduction

To move safely and efficiently through the world, animals must have the capacity to detect obstacles in their path and to do this with sufficient time to execute an avoidance manoeuvre. For visually-guided animals active in dim light, the difficulty of accurately detecting and avoiding obstacles is increased because the reduced number of available photons decreases the signal to noise ratio, making visual information less reliable (Land and Autrum 1981). Nonetheless, many visually-guided animals are active in dim light, including the bumblebee *Bombus terrestris* that, despite the limitations imposed by their apposition compound eyes that are adapted for bright daylight conditions (Nilsson and Land 2012), are capable of extending their foraging period into dawn and dusk (Steen 2017). *B. terrestris* improve their visual sensitivity in dim light, at least in part, by increasing the time over which their photoreceptors capture photons (Reber et al. 2015). This would have the effect of reducing their temporal resolution and limiting their ability to detect visual motion, a cue that is critical for controlling flight and detecting obstacles. Indeed, when landing in dim light, bumblebees extend their legs closer to the target (Baird et al. 2015) and their body posture is modified (Reber et al. 2016), suggesting that the decrease in temporal resolution does affect their behaviour. Does this also affect their ability to detect and avoid obstacles?

The aim of the present study is to answer this question by investigating how light intensity affects the ability of foraging bumblebees to detect and avoid a stationary obstacle. *B. terrestris* were trained to fly along an experimental tunnel to a feeder. An obstacle was then presented along their path and the resultant trajectories recorded.

## Materials and methods

A bumblebee hive (*Bombus terrestris*; Koppert, The Netherlands) was placed at the entrance of a 200 cm long, 30 cm high and 30 cm wide tunnel covered with netting. The bees were kept in a 18 h:7 h light:dark cycle in a controlled laboratory environment (24 °C, 32% humidity) and were trained to fly to a sugar-water feeder hidden behind a white panel at the end of the tunnel, where they were marked for individual identification. The bees were allowed to forage freely from this feeder (flying back and forth along the tunnel) for at least 2 days prior to the experiment and throughout its duration. Dimmable fluorescent lamps (BIOLUX, OSRAM GmbH, Germany) covered with white diffusion filters (LEE 252 Eight White Diffusion) illuminated the tunnel from above.

The walls and floor of the tunnel were lined with uniform 50% grey. Bees flying to the feeder were presented with two experimental conditions—(1) control and (2) obstacle—under two light intensities, 500 lx and 19 lx, chosen because they mimic the light intensities around dawn and dusk when bumblebees naturally forage and because they were similar to intensities used in similar studies with *B. terrestris* (Baird et al. 2015; Reber et al. 2016, 2015). In the control condition, no obstacle was present in the tunnel. In the obstacle condition, a 30 cm high, 5 cm diameter cylinder displaying a grey-scale dead-leaves pattern (to provide the bees with strong contrast cues and a naturalistic range of spatial frequencies, (Lee et al. 2001) was placed in the tunnel. Before each 30 min long trial commenced, the light intensity for was set and the bees allowed to forage freely for at least 30 min to allow their visual systems time to adapt to the light condition. To prevent habituation to the obstacle or its position, it was only present during the experimental trials and was placed midway between the walls at one of three locations (one location being used per trial)—25 cm before the centre, at the centre or 25 cm after the centre. Only the first three flights of an individual per trial were included in the data (although most individuals performed only one flight per trial). Each light intensity and obstacle position combination was presented in a pseudo random order. A camera (MotionBLITZ EoSens mini, Miktron GmbH, Germany) mounted above the tunnel recorded the 2 m long flights towards the feeder at 100 Hz (Fig. S1). Upon arriving at the feeder, the bee was identified by the observer and this information was added to the saved video file. At 19 lx, an infrared illuminator (TV6700, Elfa Distralec AB, Sweden) was used to improve the signal to noise ratio in the recordings without modifying the intensity of light visible to the bees.

### Data analysis and statistics

**Fig. 1:**
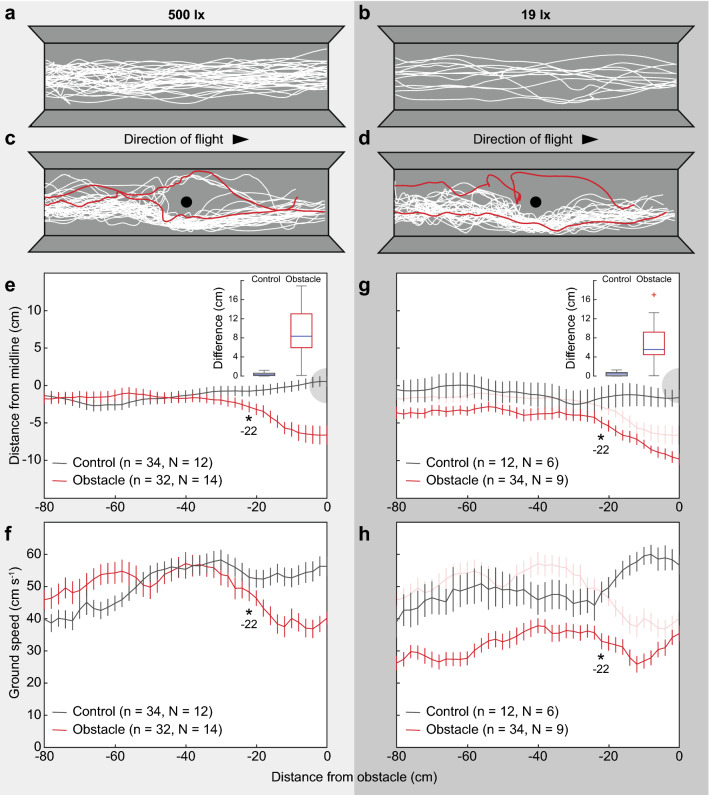
The effect of light intensity on obstacle avoidance in bumblebees. Trajectories of bees flying along a 30 cm wide experimental tunnel lined with uniform grey (control condition) at **a** 500 lx, **b** 19 lx, **c** 500 lx with an obstacle (black circle), **d** 19 lx with an obstacle (black circle). Note that the trajectories are normalised to the centre of the tunnel (**a**, **b**) or the centre of the obstacle (**c**, **d**), which varied in its location along the tunnel during the trials such that the normalised example flights shown are 160 cm long. **e** The mean lateral position or flight speed **f** of bees flying at 500 lx in either the control condition (grey data) or when the obstacle (represented by a grey shaded half-circle) was present in the tunnel (red data). **g** The mean lateral position or flight speed **h** of bees flying at 19 lx in either the control condition (grey data) or when the obstacle (represented by a grey shaded half-circle) was present in the tunnel (red data). In (**g**) and (**h**), data from the 500 lx condition with an obstacle (from (**e**) and (**f**), light red data) is included for comparison. The data in **e**–**h** represent mean values binned at 2 cm intervals, the error bars represent the standard error of the mean. Stars and values represent the distance at which the trajectories in the obstacle condition began and continued to deviate significantly from the control condition using a Students *t*-test at the 5% significance level. *N* represents the number of individuals in each condition, *n* represents the number of flights. Insets in (**e**) and (**g**) show boxplots of the difference between the lateral position at the start and end (0 cm distance from the obstacle) of each trajectory for the 500 lx and 19 lx control and obstacle data. Boxes indicate the 25th–75th percentiles, whiskers show the extent of the data, blue lines indicate the median value and red crosses indicate outliers

In each video frame, the centre of mass of the bumblebee (in *x*- and *y*-coordinates) was determined using an automated tracking program (Lindemann et al. 2005). The flight trajectories were tracked over the tunnel’s 200 cm length and normalised to the centre of the obstacle or the centre of the tunnel for the control condition. Only flights in which individual bees flew alone in the tunnel to the feeder were included in the analysis. Position data were converted from pixels to cm using a check pattern placed 15 cm above the tunnel floor (the approximate height of the flight trajectories). Ground speed was calculated by dividing the two-dimensional distance travelled between successive frames by the frame duration (0.1 s). The speed and position data for each flight were grouped in 2 cm distance bins from which a median value was calculated. The position and ground speed values at each distance step were averaged for each light intensity and the data from the trials with an obstacle were compared with the data calculated at the same relative distance for the control condition using two-tailed Student’s *t*-tests in Matlab (Mathworks, USA), as the data in each bin followed a normal distribution. A change in position or ground speed was deemed to occur when the *t*-test between the obstacle and control condition at the same distance resulted in *p* values below 0.05. Values reported in the text are mean ± std. The number of flights and individuals recorded for each condition are presented in Fig. 1. Repeated flights from the same individuals were treated as individual data points because intra- and inter-individual variation was similar (Fig. S2).

## Results and discussion

Under both light intensities, the flights in the control conditions and the obstacle were initially clustered close to but slightly to the right of the midline (Control: − 2.7 ± 4.3 cm 500 lx, − 2.4 ± 6.0 cm 19 lx; Obstacle: − 1.5 ± 4.1 500 lx, − 3.3 ± 4.1 cm 19 lx, Fig. 1a, b). When the obstacle was present, bees deviated from the start of their original path to avoid the obstacle by 9.1 ± 4.4 cm and 6.7 ± 3.9 cm in 500 lx and 19 lx, respectively. This was significantly different from the control conditions, where the deviation was 3.3 ± 3.3 cm and 4.4 ± 4.0 cm over the same distance in 500 lx and 19 lx, respectively (500 lx: *n* = 66, *p* < 0.001, 19 lx: *n* = 46, *p* = 0.007, insets Fig. 1e, g) and suggests that the bees were actively adjusting their flight path to avoid the obstacle. Interestingly, nearly all bees flew to the right of the obstacle, except for six flights to the left (5 of 34 flights in 500 lx, 1 of 34 flights in 19 lx). This may have been the result of the slight right-side tendency the bees had at the start of their trajectories. The result nonetheless raises the possibility that, when negotiating obstacles, *B. terrestris* may have a consistent side preference, which would represent an efficient strategy for minimising the risk of head-on collisions with other individuals in areas with heavy traffic, such as when they are approaching or leaving the hive or a food source.

The average speed of the flights in the control conditions was not affected by light intensity (500 lx: 50.9 ± 4.5 cm s^−1^, *n* = 34; 19 lx: 50.6 ± 4.2 cm s^−1^, *n* = 12; *p* = 0.92), contradicting the results of Reber et al. (2015), which found *B. terrestris* decreased their speed with light intensity. One major difference between these two studies is that, here, the tunnel walls did not provide strong contrast cues as they did in Reber et al. (2015). Thus, it appears as though the mechanism mediating the relationship between speed and light intensity in bumblebees is based on image motion cues because, when these cues are absent, this relationship is no longer observed.

At 500 lx, flights at the start of the tunnel were clustered close to the midline before deviating towards the right wall at a distance of 22 cm from the obstacle (*n* = 66, *p* = 0.032, Fig. 1e, Table S1). This lateral deviation also coincided with a reduction in speed (*n* = 66, *p* = 0.021, Fig. 1f, Table S1), suggesting that the bees were modifying their flight to avoid the obstacle when it subtended a visual angle of 13°. At 19 lx, the trajectories started at a similar position as those in the control, albeit slightly but not significantly tending to the right wall, before making a more distinct and consistently significant rightward deviation 22 cm from the obstacle (*n* = 46, *p* = 0.046, Fig. 1g, Table S1). Flight speed was initially slower than in the control condition and in the 500 lx obstacle condition but then made a consistent decrease at 22 cm (*n* = 46, *p* = 0.002, Fig. 1h, Table S1). As the only difference between these two conditions was the presence of the obstacle or the light intensity, these results suggest that, at 19 lx, the bees appeared to be detecting and responding to the obstacle by flying slower.

One mechanism by which *B. terrestris* may be improving the reliability of vision in dim light, in addition to increasing the temporal integration time of their photoreceptors, is spatial summation (Reber et al. 2015; Warrant 1999). This is the neural pooling of photoreceptor signals across space, which could be used to improve sensitivity in dim light but would have the effect of reducing spatial resolution (Warrant 1999). At 19 lx were responding to the presence of the obstacle when it would have subtended a horizontal angle of only 3.6° on the frontal visual field. This value closely matches the minimum spatial resolutions previously reported for *Bombus terrestris* (Chakravarthi et al. 2016: 2.4°; Dyer et al. 2008: 2.3°; Kapustjansky et al. 2010: 4°; Spaethe and Chittka 2003: 3.5°–7°; Wertlen et al. 2008: 2.5°–4°), suggesting that *B. terrestris* do not appear to significantly compromise their spatial resolution to improve their visual sensitivity in dim light. It is also possible that instead of responding to the obstacle as an object, the bees were responding to the variation in contrast of the pattern covering it. Further investigations are needed to determine the visual cues that bees rely on to detect obstacles in dim light and the relative role that spatial resolution and contrast sensitivity may play.

The deviation in position and speed in response to the obstacle occurred at a remarkably consistent distance of 22 cm, even in dim light. What mechanism might be mediating this avoidance response? One possibility is that the bees respond once the obstacle reaches a certain angular threshold on the visual field, as do locusts (Robertson and Johnson 1993) and *Drosophila* (van Breugel and Dickinson 2012). At 22 cm, the widest part of the obstacle would have subtended 13° on the frontal visual field of the bees, which is similar to the 10° threshold found for locusts but much lower than the 33° measured for *Drosophila*. One prediction of this strategy is that the size of the obstacle would affect the distance at which bees make an avoidance manoeuvre, something that remains to be tested in more detail using obstacles of different sizes.

Another possible mechanism that bumblebees may have used to determine when to initiate a deviation around the obstacle is a projected time-to-contact threshold—that is, they would respond when the time-to-contact projected from the speed of the bee and distance from the obstacle reaches a certain value. Flies use such a strategy to initiate a deceleration when landing (Wagner 1982). A time-to-contact strategy would enable the bees to respond efficiently and adaptively to obstacles even when they are flying at different speeds or when the obstacles have different widths (which would not be the case with the visual threshold hypothesis discussed above) and could ensure that there is always enough time for the visual system to detect the obstacle and to initiate a behavioural response to it. Is it possible that bumblebees use this strategy to avoid obstacles?

The data from this experiment alone are not sufficient to determine if the bees might be using a time-to-contact strategy because the object size, flight speeds and reaction distances were similar in all conditions. However, it is possible to compare the predicted time-to-contact values obtained here with those of previous studies—using a similar experimental setup—that recorded how *B. terrestris* respond to changes either in tunnel width (Baird et al. 2010) or in the optic flow presented in the lateral visual field (Linander et al. 2015). These calculations provide eight time-to-contact predictions that are remarkably similar (Table 1), with an average value of 0.33 ± 0.08 s. This value is also consistent with the time-to-contact prediction of 0.4 s, that could be made from the results of Ravi et al. (2019), which found *B. terrestris* flying at ~ 50 cm s^−1^ changed their flight at a distance of ~ 20 cm from a gap placed in their flight path (values given are approximate due to the binning method used to make these calculations). Altogether, these results provide evidence that bumblebees may indeed be using a time-to-contact strategy for obstacle avoidance. They also suggest, rather surprisingly, that the temporal summation that bees use to improve their visual sensitivity in dim light does not affect this calculation.

**Table 1:** Predicted time-to-contact thresholds for obstacle avoidance in bumblebees

Type of change presented in tunnel	Observed response to change	Ground speed at location where a response (column 2) to the change was observed (cm s^−1^)	Distance between location of observed response (column 3) and change presented in tunnel (column 1) (cm)	Predicted time-to-contact calculated from the distance at the response (column 4) divided by the speed at response (column 3) (s)
Obstacle at 500 l^a^	Reduction in ground speed	52	22	0.42
Obstacle at 500 lx^a^	Change in lateral position	52	22	0.42
Obstacle at 19 lx^a^	Reduction in ground speed	57	22	0.39
Obstacle at 19 lx^a^	Change in lateral position	57	22	0.39
Change in tunnel width from 30 to 15 cm^b^	Reduction in ground speed	77	26	0.34
Change in tunnel width from 15 to 30 cm^b^	Increase in ground speed	65	14	0.22
Change in wall pattern from check to horizontal stripe 30 cm wide tunnel^c^	Decrease in ground speed	80	18	0.23
Change in wall pattern from check to horizontal stripe 15 cm wide tunnel^c^	Change in lateral position	50	20	0.40

Using data from different studies on *B. terrestris*, this table represents calculations of time-to-contact obstacle avoidance values as predicted from bumblebees’ ground speed when a response to a change in the visual information in an experimental tunnel was first detected and the distance at which this response occurred (i.e. distance of response in cm/ground speed at response in cm s^−1^)

^a^From the present study

^b^From Baird et al. (2010)

^c^From Linander et al. (2015)

Overall, the findings of this study demonstrate that obstacle avoidance in *Bombus terrestris* is robust to changes in light intensity. The visual adaptation mechanisms that bumblebees have developed in order to forage in dim light thus appear to require little trade-off in terms of safe and efficient flight. This is also supported by an analysis of how far away from the obstacle the bees flew when they were passing by it (that is, at 0 cm from its centre), as the average lateral distance from the obstacle edge did not vary greatly between the two light intensities and was even marginally greater in dim light (500 lx: 9.4 ± 1.7 cm, *n* = 34; 19 lx: 10.3 ± 1.8 cm, *n* = 34; *p* = 0.050). Interestingly, the minimum lateral distances observed between a bee and the obstacle edge were also very similar (500 lx: 5.7 cm; 19 lx: 6.1 cm), again suggesting that *B. terrestris* can reliably determine the distance to objects under both light intensities. Considering that these bees have visual systems adapted for bright light, the findings of this study provide strong evidence that the neural mechanisms *B. terrestris* uses to improve sensitivity do not impair the precision with which they are able to detect and avoid obstacles in dim light.

## Electronic supplementary material

Below is the link to the electronic supplementary material.

Supplementary file1 (DOCX 679 kb)

## Data Availability

The data presented in this study are available in the Electronic Supplementary Material. Complete raw data are available on request.
