# Supplementary material for: Obstacle avoidance in bumblebees is robust to changes in light intensity
Source: Anim Cogn. 2020 Aug 9;23(6):1081–6. doi: 10.1007/s10071-020-01421-z (PMC7700065; doi:10.1007/s10071-020-01421-z)
Supplement: Supplementary file 1 — Supplementary file1 (DOCX 679 kb) [file 10071_2020_1421_MOESM1_ESM.docx]

Electronic Supplementary Material

hive


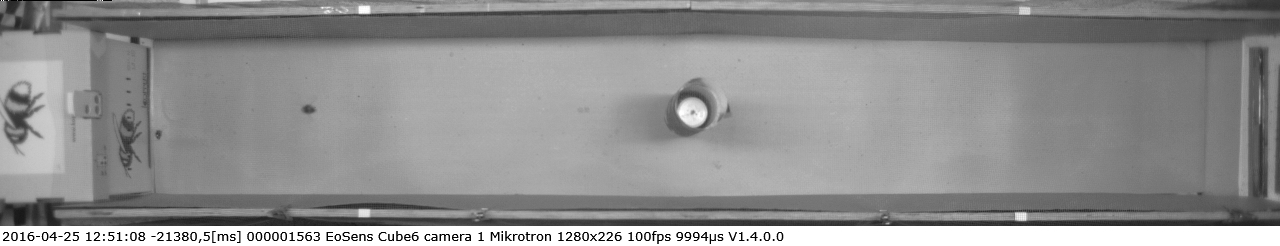


bee

obstacle

feeder

hive

Fig. S1 An image extracted from the recording of a flight showing the experimental setup. Bumblebees (centre left of image) were trained to fly along the 2 m long experimental tunnel, from the hive (far left of the image) to a ‘trough’ feeder hidden behind a white wall (far right of image). The tunnel walls were lined with grey and, in the experimental tests, a 30 cm high, 5 cm in diameter tube and covered in a texture (centre of the image) was placed either in the tunnel. For further details see Materials and Methods.

Fig. S2 Comparison of intra- and inter- individual variation in flights. Trajectories along the experimental tunnel taken by the same individual (black lines) or different individuals (coloured lines) in each of the experimental conditions. The data is normalised to the centre of the tunnel (control conditions) or the obstacle (obstacle conditions). The data show that the intra-individual variation is similar inter-individual variation.

Table S1 The results of Students t-test comparisons between conditions at different distances from the obstacle in 500 lx and 19 lx. Green values indicate where the P value is below 0.05.

| **Distance from obstacle (cm)** | **p 500 lx obstacle vs control**  lateral position  *n = 66* | **p 500 lx obstacle vs control**  speed  *n = 66* | **p 19 lx obstacle vs control**  lateral position  *n = 46* | **p 19 lx obstacle vs control**  speed  *n = 46* | **p 19 lx obstacle vs 500 lx obstacle** lateral position  *n = 66* | **p 19 lx obstacle vs 500 lx obstacle** speed  *n = 66* |
| --- | --- | --- | --- | --- | --- | --- |
| **-80** | 0.6066 | **0.0100** | 0.0822 | **0.0220** | 0.1255 | **0.0001** |
| **-78** | 0.6265 | **0.0043** | 0.0516 | **0.0070** | 0.0751 | **0.0002** |
| **-76** | 0.8487 | **0.0014** | 0.0860 | **0.0208** | 0.0911 | **0.0003** |
| **-74** | 0.9840 | **0.0081** | 0.0720 | **0.0150** | 0.0636 | **0.0001** |
| **-72** | 0.8728 | 0.0627 | 0.0571 | **0.0030** | 0.0675 | **0.0002** |
| **-70** | 0.5806 | **0.0308** | 0.0816 | **0.0010** | 0.0994 | **0.0000** |
| **-68** | 0.5201 | **0.0234** | 0.0548 | **0.0003** | 0.1211 | **0.0000** |
| **-66** | 0.4102 | **0.0056** | **0.0335** | **0.0003** | 0.0807 | **0.0000** |
| **-64** | 0.4196 | **0.0015** | **0.0294** | **0.0001** | 0.0818 | **0.0000** |
| **-62** | 0.4306 | 0.0101 | **0.0279** | **0.0001** | 0.0841 | **0.0000** |
| **-60** | 0.3201 | 0.1224 | **0.0199** | **0.0000** | **0.0413** | **0.0000** |
| **-58** | 0.2339 | 0.0111 | **0.0384** | **0.0002** | 0.0510 | **0.0000** |
| **-56** | 0.2964 | 0.0145 | 0.0662 | **0.0004** | 0.0577 | **0.0000** |
| **-54** | 0.4637 | **0.0472** | 0.1193 | **0.0069** | 0.1022 | **0.0001** |
| **-52** | 0.5968 | 0.2023 | 0.1482 | **0.0056** | 0.0938 | **0.0004** |
| **-50** | 0.7482 | 0.3698 | 0.1133 | **0.0047** | 0.0642 | **0.0003** |
| **-48** | 0.8804 | 0.4010 | 0.0920 | 0.0056 | 0.0738 | **0.0001** |
| **-46** | 0.9199 | 0.2981 | 0.0764 | 0.0111 | **0.0420** | **0.0000** |
| **-44** | 0.9014 | 0.5132 | 0.0807 | 0.0181 | **0.0320** | **0.0000** |
| **-42** | 0.7239 | 0.6375 | 0.1177 | **0.0477** | **0.0446** | **0.0000** |
| **-40** | 0.6612 | 0.6030 | 0.1438 | 0.0980 | **0.0491** | **0.0000** |
| **-38** | 0.6541 | 0.7815 | 0.1770 | 0.0545 | **0.0406** | **0.0000** |
| **-36** | 0.5330 | 0.8590 | 0.2053 | **0.0255** | **0.0384** | **0.0000** |
| **-34** | 0.3499 | 0.9115 | 0.3490 | **0.0240** | 0.0942 | **0.0000** |
| **-32** | 0.2131 | 0.9115 | 0.3749 | **0.0327** | 0.0613 | **0.0000** |
| **-30** | 0.1972 | 0.5425 | 0.4050 | 0.0644 | 0.0530 | **0.0001** |
| **-28** | 0.1320 | 0.5035 | 0.3860 | **0.0434** | 0.0858 | **0.0001** |
| **-26** | 0.0969 | 0.0951 | 0.2722 | **0.0291** | 0.0792 | **0.0015** |
| **-24** | 0.0561 | 0.0733 | 0.1727 | 0.0691 | 0.1157 | **0.0006** |
| **-22** | **0.0316** | **0.0206** | **0.0458** | **0.0018** | 0.0521 | **0.0001** |
| **-20** | **0.0138** | **0.0066** | **0.0168** | **0.0003** | **0.0434** | **0.0001** |
| **-18** | **0.0076** | **0.0021** | **0.0035** | **0.0000** | **0.0195** | **0.0021** |
| **-16** | **0.0008** | **0.0014** | **0.0008** | **0.0000** | **0.0283** | **0.0127** |
| **-14** | **0.0001** | **0.0008** | **0.0009** | **0.0000** | 0.0799 | **0.0096** |
| **-12** | **0.0000** | **0.0009** | **0.0003** | **0.0000** | 0.1768 | **0.0066** |
| **-10** | **0.0000** | **0.0044** | **0.0000** | **0.0000** | 0.0853 | **0.0010** |
| **-8** | **0.0000** | **0.0007** | **0.0000** | **0.0000** | 0.0707 | **0.0040** |
| **-6** | **0.0000** | **0.0001** | **0.0000** | **0.0000** | 0.0849 | 0.0849 |
| **-4** | **0.0000** | **0.0002** | **0.0000** | **0.0000** | 0.0795 | 0.1321 |
| **-2** | **0.0000** | **0.0004** | **0.0000** | **0.0000** | 0.0535 | 0.2380 |
| **0** | **0.0000** | **0.0006** | **0.0000** | **0.0000** | **0.0381** | 0.1373 |
